# Effectiveness of adolescent suicide prevention e-learning modules that aim to improve knowledge and self-confidence of gatekeepers: study protocol for a randomized controlled trial

**DOI:** 10.1186/1745-6215-15-52

**Published:** 2014-02-08

**Authors:** Rezvan Ghoncheh, Ad JFM Kerkhof, Hans M Koot

**Affiliations:** 1 Department of Clinical Psychology and the EMGO + Institute for Health and Care Research, Faculty of Psychology and Education, VU University Amsterdam, Van der Boechorststraat 1, 1081 BT, Amsterdam, The Netherlands; 2 Department of Developmental Psychology and the EMGO + Institute for Health and Care Research, Faculty of Psychology and Education, VU University Amsterdam, Van der Boechorststraat 1, 1081 BT, Amsterdam, The Netherlands

**Keywords:** Adolescents, E-learning, Gatekeepers, Modules, Online, Prevention, Professionals, RCT, Suicide, Training

## Abstract

**Background:**

Providing e-learning modules can be an effective strategy for enhancing gatekeepers’ knowledge, self-confidence and skills in adolescent suicide prevention. The aim of this study was to test the effectiveness of an online training program called *Mental Health Online* which consists of eight short e-learning modules, each capturing an important aspect of the process of recognition, guidance and referral of suicidal adolescents (12–20 years). The primary outcomes of this study are participant’s ratings on perceived knowledge, perceived self-confidence, and actual knowledge regarding adolescent suicidality.

**Methods/Design:**

A randomized controlled trial will be carried out among 154 gatekeepers. After completing the first assessment (pre-test), participants will be randomly assigned to either the experimental group or the waitlist control group. One week after completing the first assessment the experimental group will have access to the website *Mental Health Online* containing the eight e-learning modules and additional information on adolescent suicide prevention. Participants in both conditions will be assessed 4 weeks after completing the first assessment (post-test), and 12 weeks after completing the post-test (follow-up). At post-test, participants from the experimental group are asked to complete an evaluation questionnaire on the modules. The waitlist control group will have access to the modules and additional information on the website after completing the follow-up assessment.

**Discussion:**

Gatekeepers can benefit from e-learning modules on adolescent suicide prevention. This approach allows them to learn about this sensitive subject at their own pace and from any given location, as long as they have access to the Internet. Given the flexible nature of the program, each participant can compose his/her own training creating an instant customized course with the required steps in adolescent suicide prevention.

**Trial registration:**

Netherlands Trial Register NTR3625

## Background

Suicide is the second leading cause of death among adolescents in the Netherlands, affecting an average of 50 adolescents every year [1]. In addition, each year, 11.2% of Dutch adolescents have suicidal thoughts and 6.6% attempt suicide or engage in deliberate self-harm [2]. Ethnic minorities, especially Hindustan-Surinam and Turkish adolescents more often report suicidal thoughts and attempts compared to their Dutch peers [3,4]. This is alarming since 23.5% of the Dutch adolescent population (0–20 years) consists of ethnic minority groups, the three biggest ethnic minority groups in the Netherlands being of Moroccan (15.6%), Turkish (14.1%), and Surinamese (9.8%) origin [1]. Despite fluctuations, the Dutch suicide numbers have stayed stable over the past decade [1], and until recently the state of available suicide prevention programs was still unclear. Therefore, in 2009 the Dutch government assigned a group of experts the task of preparing an overview of all the suicide prevention and intervention programs in the Netherlands [5]. This inventory led to several recommendations on how to improve suicide prevention in the Netherlands. One of the advices was to educate gatekeepers involved in adolescent suicide prevention with special attention for ethnicity.

In this paper gatekeepers are defined as professionals who, due to their profession, come into contact with adolescents who may be at risk for suicide, such as school staff, youth nurses, and primary health care providers [6,7]. According to several sources, these professionals can play an essential role in early recognition, guidance, and referral of suicidal adolescents [6,8]. Moreover, research conducted in this area shows that gatekeeper training is a promising tool in improving suicide prevention [9-13]. If trained properly, gatekeepers can be among the first to perceive changes in the behavior and attitude of the adolescent in need. Thus, effective training of gatekeepers seems a worthwhile investment. However, two important factors tend to keep gatekeepers from attending face-to-face training sessions on suicide prevention. First, suicide is a topic surrounded by social stigma and taboo [6] which makes suicide a sensitive subject for potential gatekeepers to address in general. Second, most gatekeepers have busy schedules and have limited time and resources to attend face-to-face training sessions [13,14]. Notwithstanding these issues, the Dutch government is giving gatekeepers more responsibilities when it comes to the welfare of children and adolescents. Thus, gatekeepers are required to receive training in different mental health matters that are common among adolescents, while this usually constitutes a task addition to their main duties. As a result, there is a need for time and resource-saving methods of training for these gatekeepers.

As an answer to this need, in 2011 the departments of Developmental Psychology and Clinical Psychology at VU University Amsterdam started a government-funded program called *Mental Health Online*, which aims to enhance suicide prevention among adolescents (12–20 years). The main objective of this program is to educate gatekeepers in adolescent suicide prevention through e-learning modules, defined as packed pieces of information that transfer knowledge to the learner through a web-based structure [15]. The online character of the training is expected to create a low threshold to educate gatekeepers on this topic and is time-saving at the same time. Furthermore, recent data show that at least 94% of the Dutch households have access to the Internet and 86% of Internet users go online daily or almost daily [1]. Thus, by offering the training online, several practical barriers that prevent gatekeepers from attending face-to-face training on adolescent suicide prevention might be overcome.

### Aims and hypotheses

The aim of this study is to develop and test the effectiveness of e-learning modules that educate gatekeepers in adolescent suicide prevention. It is expected that the e-learning modules will improve the knowledge of gatekeepers regarding adolescent suicidality (defined as suicidal behavior, i.e., ideation and actions) and that the gatekeeper’s self-confidence in interacting with suicidal adolescents will increase.

## Methods

### Study design

This study is a randomized controlled trial with two arms: an experimental group and a waitlist control group. Participants from both groups will complete three assessments: at baseline (pre-test, T₀), four weeks after completing the first assessment (post-test, T₁), and 12 weeks after T₁ (follow-up, T₂). Participants will be assigned to one of the two groups (experimental or waitlist control group) after completing the baseline assessment. The difference between the two groups is that participants in the experimental group will have access to the e-learning modules and additional information on the website *Mental Health Online* (http://www.MentalHealthOnline.nl) seven days after completing the first assessment, while participants in the waitlist control group have to wait until the follow-up assessment is completed.

The assessments consist of three questionnaires which measure the perceived knowledge, the perceived self-confidence, and the actual knowledge of participants in both groups regarding adolescent suicidality. By comparing the questionnaire information obtained from the two groups during the three assessments it is tested whether the perceived knowledge, the perceived self-confidence, and the actual knowledge of participants in the experimental group improves more than in participants in the waitlist control group after gaining access to the e-learning modules and additional information on the website.

The study protocol for *Mental Health Online* has been approved by the Medical Ethics Committee of the VU University Medical Centre Amsterdam (registration number 2009/328).

### Participants

All Dutch speaking professionals who work with adolescents are eligible to participate in this study. Inclusion criteria are that gatekeepers should i) be 18 years and older, ii) work regularly with adolescents from 12 to 20 years and be directly responsible for their (mental) healthcare, and iii) they must have access to the Internet.

Although all gatekeepers who meet the inclusion criteria are allowed to participate in the study, the following groups of professionals have been identified as the main target groups of this study: members of mental healthcare teams in schools, youth nurses, and mental healthcare employees.

### Interventions

The experimental group will have access to the restricted area of the website *Mental Health Online* (http://www.MentalHealthOnline.nl) which is especially designed for the participants of this study and which contains eight e-learning modules and additional information on adolescent suicide prevention. Each module captures an important aspect of the process of recognition, guidance, and referral of adolescent suicidality (Additional file 1). The modules are based on the Question, Persuade and Refer (QPR) Gatekeeper Training which has gained worldwide recognition when it comes to training gatekeepers in suicide prevention. The QPR model has been created by Paul Quinnett in 1995 [8]. It is composed of several essential steps in suicide prevention: early recognition of the warning signs associated with suicidality, asking direct questions regarding the suicidal thoughts, feelings, and plans of the person who might be in a suicidal state, and starting a conversation persuading the person to accept a referral for help. Research regarding the effectiveness of gatekeeper training in general is limited, but the available research on the QPR gatekeeper training is promising. Gatekeepers receiving the QPR training show improvements in knowledge about suicide prevention, skills, and self-efficacy [16-18].

The modules have been developed by the researchers in this study in collaboration with national and international experts in suicide prevention. The modules are presented using Adobe Presenter 7 software to convert PowerPoint slides into e-learning modules. Each module takes up to 10 minutes to complete and contains voice-over, cases, and quizzes. The modules have been designed in a way that the participant controls every aspect of the module such as skipping or replaying a slide and switching the voice-over on or off. Moreover, by offering the program in eight separate modules gatekeepers have the opportunity to compose a custom-made training for themselves based on their own personal experiences and needs. However, since the amount of modules each participant follows could influence the scores on the questionnaires, a user-tracking system has been enabled on the website. With this system, data will be collected regarding the amount of modules each participant follows between the pre- and post-test, and post-test and follow-up.

Besides the modules, participants also will have access to additional information regarding adolescent suicidality on the website, such as literature and documentaries on suicidality written in Dutch, but also links to other relevant websites that contain information on (adolescent) suicidality. Furthermore, participants will have access to an online discussion board on the website which gives them the opportunity to exchange thoughts with each other, but also to consult a group of experts regarding adolescent suicidality.

### Primary outcome measures

The primary outcomes are i) participants’ ratings on perceived knowledge regarding adolescent suicidality, ii) perceived self-confidence when interacting with suicidal adolescents, and iii) actual knowledge regarding adolescent suicidality.

Since this is the first time e-learning modules on adolescent suicide prevention for gatekeepers have been developed and in order to test their effectiveness, the authors have composed three questionnaires to test the three primary outcomes of this study. This was done in collaboration with adolescent suicide prevention experts involved in this project. In order to test the first and the second primary outcome measures, two short questionnaires have been developed consisting of statements to be endorsed on a three-point Likert scale (0 = disagree, 1 = partially agree, 2 = agree).

The questionnaire measuring perceived knowledge consists of nine items. The first item is a general statement about the overall knowledge of the participants when it comes to adolescent suicide prevention (‘I have sufficient knowledge about the process of recognition, guidance, and referral of suicidal youth’), and the remaining eight statements each capture the essence of one of the eight modules offered. For example, the fifth module focuses on how to engage in a conversation with a suicidal adolescent and the statement measuring the perceived knowledge regarding this module is ‘I have sufficient knowledge to engage in a conversation with a suicidal adolescent’. The questionnaire measuring perceived self-confidence consists of 16 items, each capturing the required skills and attitudes in adolescent suicide prevention which are addressed in the eight modules. Examples of the statements are ‘I can adequately provide first aid to a young person who has attempted suicide’ and ‘I can make a distinction between my duties and those of a therapist’. During the three assessments of the effectiveness study participants receive the same two questionnaires.

To assess the last primary outcome, actual knowledge, six cases have been developed. Each case starts with a photo of and some characteristics about an imaginary adolescent (name, age, and education are added), and is followed by three informational segments. The segments provide information about the background, friends, and family circumstances of the adolescent. After reading each case, the participant is asked to answer several questions on potential suicidality of which two are general questions (yes/no) and eight questions with multiple choice answers of which one is correct, covering the content of each of the eight e-learning modules. The number of questions the participant has to answer depends on the answers given on the general questions. During each assessment (T₀, T₁, T₂) participants will be presented with two new cases.

### Secondary outcome measures

The secondary outcome is the satisfaction of the gatekeepers regarding the design of the e-learning modules. During the second assessment, participants from the experimental group are asked to complete a short evaluation questionnaire. In this questionnaire, characteristics of the e-learning modules are evaluated using 34 questions on expectations (2 items), relevance (2 items), design (5 items), interaction (2 items), cases and quizzes (11 items), overall satisfaction (6 items), website (5 items), and final commentary (1 item).

The questions are answered using the following format: a four-point Likert scale (17 items), multiple choice (3 items), rating (2 items), yes/no (4 items), and multiple responses (1 item). Seven of the questions are optional open questions which give the participant the opportunity to elaborate on their previous answers. Based on the answers the e-learning modules will be adjusted for implementation.

In addition, an attempt is made to improve the skills of the gatekeepers by providing them with frameworks, tips, and information in the e-learning modules. Due to privacy issues it is not possible to measure changes in this particular area as monitoring each gatekeeper, including an overview of false and correct referrals, would be required. However, participants from the experimental group are asked to rate several general statements on a four-point Likert scale as part of the evaluation questionnaire. One of these statements concerns the perceived gain in skills (‘By following the e-learning modules I have gained more skills in interaction with suicidal adolescents’). By asking the participants to rate this particular statement an overall indication of the impact of the e-learning modules on the skills of the participants can be obtained.

### Sample size

Sample size calculation was performed using STATA version 8.2. Based on a power of 0.80 with a two-sided α of 0.05 and assuming a mean standardized effect size of (Cohen’s *d*) 0.45 a total of 154 participants are needed, 77 participants in the experimental group and 77 participants in the waitlist control group. Due to a lack of studies in this area, the standardized effect size used to calculate the sample size for this study is not based on previous studies or a pilot study. As an alternative, a moderate effect size has been estimated.

### Randomization

Every time a participant completes the baseline assessment, the researcher sends an e-mail to an independent external data manager to randomize this person to one of the two groups. The data manager uses block randomization in IBM Statistics SPSS and reports the outcome to the researcher by e-mail.

### Assessments

Within four days after completing the baseline assessment the participants receive an e-mail from the researcher regarding the group they are assigned to and are informed on which day they will receive the second assessment. Additional to this information, those in the experimental group receive a personal username and password to log in to the website *Mental Health Online* and are informed regarding the exact period (14 days) during which they have access to the website. An instruction to the website is also attached to this e-mail.

Four weeks after completing the first assessment participants from both groups receive the second assessment (post-test), and 12 weeks after completing the second assessment the third assessment (follow-up) will be sent to the participants.

The three assessments which consist of the three questionnaires (including the evaluation questionnaire) are sent to the participants as a complete package online, and data collection is performed online.

Participants from the experimental group will regain access to the modules and additional information on the website after completing the second assessment until one week prior to the last assessment. This way, participants from the experimental group can return to the website to refresh their knowledge, follow modules they did not follow during the 14 days they had access to the website, and use the additional information and the online discussion board on the website. The reason behind making the website accessible during this relatively long period has to do with the online character of the program. By providing the modules online, we can create a low threshold to the training which is accessible to gatekeepers 24/7.

**Figure 1:**
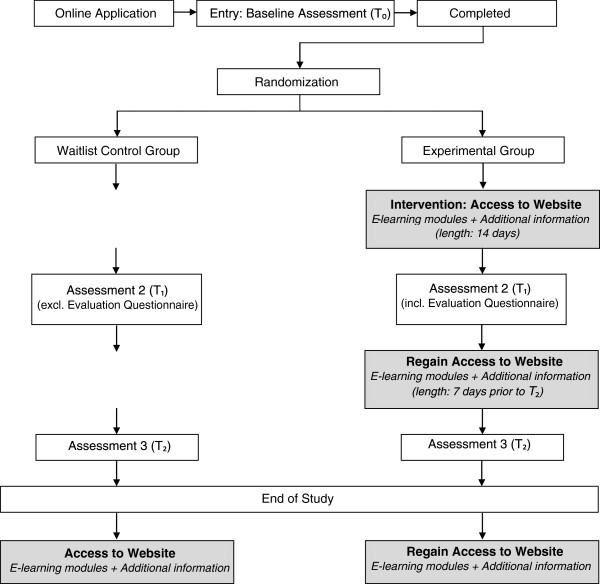
Trial flowchart.

After completing each assessment, the researcher sends a personal email to the participant in which information regarding the next assessment is provided. Furthermore, participants will receive reminders by email to complete the assessments. Participants will receive weekly reminders for the first and the third assessment. However, since it is important that the second assessment is completed immediately after accessing the website, reminders to finalize this assessment will be sent every other day if necessary. After completing the third and final assessment, participants from the waitlist control group will receive an email with a personal username and password for the website, including instructions for the website. The trial flowchart of this study is given in Figure 1.

### Statistical analysis plan

The internal consistency reliability of the three questionnaires will be estimated with Cronbach’s alpha and exploratory factor analysis will be used to check for dimensionality.

To test whether the knowledge of gatekeepers and the gatekeeper’s self-confidence increases after gaining access to the website, mean differences of the two groups (experimental and waitlist control) on the three questionnaires will be compared for the three assessment points.

Both analysis of covariance (ANCOVA) and hierarchical linear modeling (HLM) are suitable methods to test the hypotheses of this randomized pre-test, post-test, and follow-up design [19].

An ANCOVA in which pre-test is used as a covariate can be used to test for group differences from pre-test to post-test, from pre-test to follow-up, and from post-test to follow-up. By including the pre-test as a covariate in the model, more statistical power and statistically accurate confidence intervals of the differences of means between the experimental group and the waitlist control group will be gained [19]. Missing data will be dealt with by multiple imputation [20-22]. Alternatively, HLM may be used in which individual growth curves over time are integrated in the model [19,23]. HLM assumes that data are missing at random [24].

The data from the evaluation questionnaire, which is only completed by participants from the experimental group, will be analyzed using descriptive statistics. All analyses will be carried out on intention-to-treat sample.

### Recruitment

Several recruitment strategies are going to be used to recruit gatekeepers to participate in this study. First, education partnerships representing high schools in different regions and community colleges are going to be contacted by e-mail. In this e-mail, a brief summary about the study will be presented and a flyer about the research will be attached. Further, a link to the website *Mental Health Online* is included, where up-to-date information regarding the study is available. The partnerships are asked to distribute this e-mail among their members. Second, informational websites targeting gatekeepers are going to be contacted and will be asked to put a brief summary about this study on their website with a link to the website *Mental Health Online* for those interested in this study. Third, study coworkers will recruit participants during different seminars and conferences visited by gatekeepers. Fourth, a press release will be distributed by VU University Amsterdam containing information about this research and ways of contacting the study’s researchers. Finally, social media, such as Twitter and Facebook, will be used to recruit gatekeepers. Moreover, all institutes that are interested are given the opportunity to invite the researcher performing the study to give a short presentation at their institute about this study. The recruitment starts in September 2012 and the inclusion continues until September 2013.

All gatekeepers who are willing to participate in this study are required to send an e-mail to info@mentalhealthonline.nl containing the following information: name, position, name of the institute the gatekeeper is employed at, and their email address. The application instructions are given on the website, in the recruitment flyer, and at the end of each presentation given on location by the researcher. After enrollment each participant receives a confirmation email containing a brief summary about the study: purposes, procedure, contact information of the researcher, and the date on which the questionnaire for the first assessment will be sent.

## Discussion

This randomized controlled trial will test the effectiveness of e-learning modules on adolescent suicide prevention in promoting knowledge and self-confidence in gatekeepers dealing with this subject.

The use of e-learning modules for adolescent suicide prevention is expected to be beneficial for gatekeepers for several reasons. First, the subject of suicide is surrounded with stigma and taboo which can hamper gatekeepers to actively seek information on this topic through participation in face-to-face training courses. By offering the training online, gatekeepers will have access to information on the required steps in adolescent suicide prevention from their own home or workplace. Second, gatekeepers, especially those working in schools, do not always have the required resources and time to participate in face-to-face training sessions. The online character of the program’s modules gives gatekeepers the opportunity to have access to the program 24/7 from any given location. By offering the program in divided short modules, gatekeepers have the opportunity to compose their own personal training based on their own pre-existing knowledge, experience, and needs. This way, they are no longer obliged to attend an extensive training while they may only be interested in a small segment of the training offered. Third, these e-learning modules can be developed and maintained with limited resources, which allows the training to be offered for free. Since the modules are offered online, gatekeepers from all over the nation can have access to the program immediately.

The main limitation of this study has to do with the questionnaires used during the three assessments. Since this is the first time e-learning modules on adolescent suicide prevention have been developed for gatekeepers and are being tested in a randomized controlled trial, three questionnaires had to be developed in order to measure the three outcome measurements of this study. Thus, the questionnaires have not yet been validated and the current trial is the first to provide information on the usefulness of the questionnaires. If this line of research will gain popularity and more randomized controlled trials will be carried out in this area, it is recommended that validated questionnaires should be developed and used to test the effectiveness of e-learning modules on adolescent suicide prevention by gatekeepers.

## Trial status: ongoing

The recruitment for this study started in September 2012 and the inclusion continues until September 2013.

## Competing interests

The authors declare that they have no competing interests.

## Authors’ contributions

RG created the website http://www.MentalHealthOnline.nl, the eight e-learning modules (incl. voice-over) of the program Mental Health Online, and developed the three questionnaires of this study (perceived knowledge, perceived self-confidence and actual knowledge). AK and HK provided feedback for improvement of the e-learning modules and the three questionnaires. RG authored this protocol paper, AK and HK provided feedback for improvement of this manuscript. All authors read and approved the final manuscript.

## Supplementary Material

Additional file 1 The eight e-learning modules of the *Mental Health Online* program. Click here for file
