# Supplementary material for: Effectiveness of adolescent suicide prevention e-learning modules that aim to improve knowledge and self-confidence of gatekeepers: study protocol for a randomized controlled trial
Source: Trials. 2014 Feb 8;15:52. doi: 10.1186/1745-6215-15-52 (PMC3923552; doi:10.1186/1745-6215-15-52)
Supplement: Additional file 1 — The eight e-learning modules of the Mental Health Online program. [file 1745-6215-15-52-S1.pdf]

Additional file 1. The Eight E-learning Modules of the Mental Health Online Program.

Module	Aim
1. Suicidality among adolescents	Gives an overall introduction to the subject of adolescent suicidality and provides the gatekeeper with valuable information on this topic
2. Risk factors	The most common and important risk factors which underlie adolescent suicidality are discussed
3. Ethnicity	Discusses the relationship between ethnicity and adolescent suicidality, and provides the gatekeeper with the required skills when interacting with adolescents from ethnic minority groups
4. Recognition of suicidality	The most important and common warning signs associated with adolescent suicidality are discussed
5. Conversation with the suicidal adolescent	The gatekeeper is provided with a framework and the skills required when engaging in a conversation with a suicidal adolescent
6. Conversation with the parents	Important steps and skills are provided regarding when and how to engage in a conversation with the parents of a suicidal adolescent
7. Suicide first-aid	Discusses how to give first-aid to an adolescent who has attempted suicide
8. Care and aftercare (for schools)	A module specially designed for schools which offers them guidelines regarding the process of care and aftercare when an adolescent commits or attempts suicide
